# Signal growth in a pure time-modulated transmission line and the loss effect

**DOI:** 10.1098/rsos.240569

**Published:** 2024-11-13

**Authors:** Mohamed F. Hagag, Thomas R. Jones, Karim Seddik, Dimitrios Peroulis

**Affiliations:** ^1^ Electronic Engineering Department, Military Technical College, Cairo 11766, Egypt; ^2^ Electronics and Communications Engineering Department, American University in Cairo, Cairo 11835, Egypt; ^3^ Elmore Family School of Electrical and Computer Engineering, Purdue University, West Lafayette, IN 47907, USA

**Keywords:** signal growth, time-modulation, transmission line, amplification

## Abstract

We present the first comprehensive study for signal growth in transmission lines (TL) with purely time-modulated characteristic impedance $Z_{o}$ (infinite superluminality). This study pioneers the investigation into the effects of varying the cell’s electrical length and the impact of loss on momentum bandgaps and amplification levels. It also thoroughly examines how time-modulated transmission line truncation by a static load influences the sensitivity of amplification gain to the relative phase between the incoming signal and modulation, comparing these findings with the case of parametric amplification. Varying $Z_{o}$ is accomplished by loading TLs with a sinusoidally time-modulated capacitor (TMC). The study starts with a simple lumped model cell to facilitate understanding of the phenomena. Following this, transmission lines are introduced, and the effects of incorporating loss are examined. To accomplish this, three models are investigated: a lossless L-C TL lumped model loaded with a shunt lossless TMC and a TL loaded with a shunt lossless and lossy TMC. Dispersion diagrams are plotted and momentum bandgaps are identified at a modulation frequency double the signal frequency. Within the momentum bandgap, only imaginary frequencies are found and correlated to momentum bandgap width and signal growth level. Signal growth is confirmed using harmonic balance and transient simulations, and the results are consistent with the dispersion diagram outcomes.

## 1. Introduction

Temporal modulated media is an incoming technique that unleashes enormous opportunities for artificial electromagnetic media [1–3]. It is realized by varying the media characteristics periodically with time following a specific modulation waveform. The interest in engineering artificial media and materials with time-modulated properties has recently increased. One attractive proposed property of time-modulated media is magnetless non-reciprocity [4,5]. Many magnetless non-reciprocal components are presented in the literature, such as filters, circulators and power dividers [6–10]. The non-reciprocity is created when a subcomponent property (unit cell) is time modulated with a speed lower than the light speed (subliminal modulation) with a sufficient successive phase shift, creating a spatio-temporal modulated component [11–13]. Other interesting applications are proposed, such as creating an effective magnetic field for photons [14] and synthetic dimensions [15,16].

More interesting phenomena are discovered when modulating media properties in time with infinite superluminality, which requires high modulation speed, as defined in Caloz & Deck-Leger [17,18]. This was first proposed as a photonic time crystal or generally artificial time crystal [19,20]. The conventional photonic crystal is an artificial periodic structure at which the signal wave number (K) is engineered through space to create a frequency bandgap; within the frequency bandgap, the signal is forbidden to propagate [21,22]. As an analogy to photonic crystals, artificial time crystals are created when periodic structure properties are modulated in time and kept uniform in space (pure-time modulation). With a sufficient time modulation speed, bandgaps in momentum are created inside which the photonic time crystals possess a non-Hermitian nature. Within the momentum bandgap, only complex frequencies exist, which causes the signal to be amplified exponentially in the momentum bandgap [1,15], in contrast to the signal decay in the frequency bandgap [21,22]. To realize a momentum bandgap in an artificial time crystal, its properties must be modulated in time with a speed higher than the signal propagation speed inside artificial time crystals (superluminal regime).

Signal growth in a media with time-modulated parameters has been discussed in many flavours in the literature. In [23,24], Martínez-Romero & Halevi and Koutserimpas *et al*. discussed wave amplification in a slab with a time-modulated permittivity and/or permeability; they showed that a parametric resonance exists at a 0.5 signal/modulation frequency ratio in a continuous time-modulated media. In [25], Pendry *et al*. showed that an input continuous signal can be amplified as a pulsed signal using space-time luminal grating. Due to the variation of grating moving velocity. Within the time cycle, the media jumps between subluminal and superluminal regimes, causing a pulsed amplification profile. In [26], utilizing non-Foster elements, Pacheco-Peña *et al*. proved that a rapid change in a media’s ϵ(t) causes the field to be frozen with a growing amplitude. Stepping back to the earlier permittivity allows the wave to propagate again with the original frequency. By utilizing spatio-temporal modulation, Yang *et al*. introduced a cascaded phase-matching mechanism to amplify signals by transferring the energy from the fundamental mode to higher harmonics [27]. Finally, amplification in a media with a randomly changing media permittivity is studied as a disordered photonic time crystal in Sharabi *et al*. [28]. On the other hand, most of the introduced work in the literature does not cover realizable structures that can achieve time-modulation of the media in the superluminal regime. The main challenge is to find a medium with controllable and highly responsive electrical properties. Realizing such media in the microwave regime is often more attainable. This regime has adaptive components such as varactors and photodiodes; the change in their properties may be able to catch up with the modulation signal. To the best of the authors’ knowledge, Wang *et al*. presented the first realized photonic time crystal in metasurface configuration [29]. They loaded a slab of a metasurface with modulated varactors to amplify the surface wave. Moreover, in Wang *et al*. [30], their work concluded with a realizable structure with future plans to fabricate and measure.

Due to the importance of transmission lines (TL) as essential elements in building more complex circuits and systems, studying TLs with time-modulated parameters in their lumped and distributed forms is an important step in exploring new circuits with exotic properties, from radio frequencies to THz wavelengths. By spatio-temporally modulating transmission lines in the subluminal regime, magnetless non-reciprocal propagation is achieved, which allows the realization of many important magnetless components such as isolators, circulators and non-reciprocal filters [6–10,31,32]. Moreover, scattering form from time-modulated TL loads has been studied in Malléjac & Fleury [33] However, most of the presented work is achieved by doing parametric simulations by varying the modulation frequency and successive phase shifts between the cells until the functionality is achieved. To the best of the authors’ knowledge, this manuscript presents the first comprehensive study of signal growth in TL with a time-modulated characteristic impedance with infinite superluminality as a step in realizing signal amplification in the microwave regime. Additionally, the study investigates, for the first time, the effects of varying the cell’s electrical length and the impact of loss on momentum bandgaps and amplification levels. Additionally, the study thoroughly examines the influence of time-modulated TL truncation by a static load on the sensitivity of amplification gain to the relative phase between the incoming signal and modulation, comparing it with the case of parametric amplification. Modulating the characteristic impedance is achieved by loading the TLs with time-modulated capacitors (TMC). Such capacitors can be realized using high-speed varactors, Schottky or photodiodes integrated with fixed capacitors. The integration between solving the eigenvalue problem and the usage of commercial software simulations to design time-modulated circuits in a superluminal regime, which can be extended to other regimes (subluminal), is presented. The study begins with a simple lumped model cell to aid in understanding the phenomena. Subsequently, transmission lines are introduced, and the impact of adding loss is explored. The dispersion diagram is plotted, and the areas of the momentum bandgap are defined. To investigate the circuit performance within the momentum bandgap, S-parameter [34], transient and harmonic balance [35] simulations in Keysight Advanced Design System software (ADS) are performed on nine unit cells.

## 2. Signal growth in a transmission line with a time-modulated capacitor

**Figure 1.**
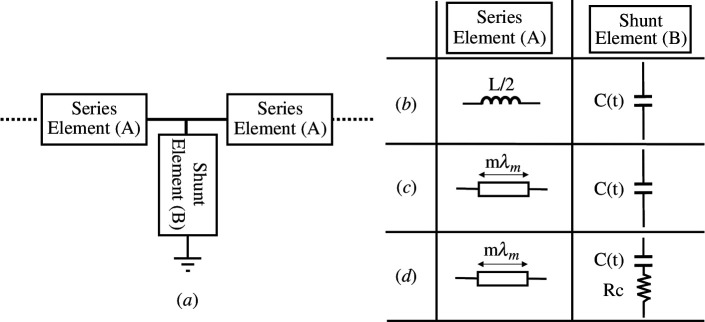
(*a*) General symmetric unit cell of an ideal transmission line. Series element (A) and shunt element (B) considering (*b*) a unit cell of a lossless lumped model of a transmission line with a time-modulated capacitor (TMC), a unit cell of a lossless TL with a length of $m\lambda_{m}$ and 90Ω characteristic impedance loaded with (*c*) lossless time-modulated capacitor, (*d*) lossy TMC.


This section discusses the signal growth in a TL loaded with a TMC. The unit cell of the loaded TL, depicted in figure 1*a*, features a T-shaped symmetric design composed of two distinct elements: series and shunt. By adjusting the shunt element (B) and the series element (A), three models are developed and analysed, as illustrated in figure 1. In this study, the capacitance is modulated around its nominal value in a sinusoidal pattern following

(2.1) $$C\left(\omega ,t\right)=C_{o}(\omega )\left(1+M_{D}\cos(\omega_{M}t+\varphi_{M}\right)),$$

where $M_{D}$ and $\varphi_{M}$ are the modulation depth and phase, respectively. $C_{o}(\omega )$ is the capacitance nominal value. The detailed construction of the dispersion equation for all discussed models is provided in the electronic supplementary material.

### 2.1. Signal growth in a lossless transmission line lumped model with time-modulated capacitor

**Figure 2.**
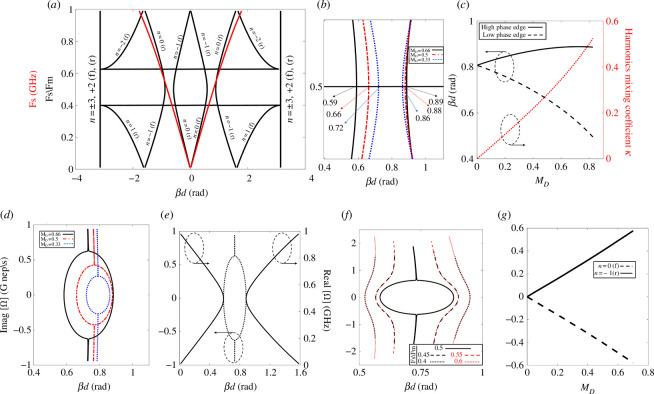
(*a*) Dispersion diagram of the unit cell in figure 1*b* with L=12.5 nH, $C_{o}=5$ pF and $M_{D}=0.66$. In the case of no modulation, the diagram only reduces to the red curves. (*b*) Close-up look of the momentum bandgap area at different values of $M_{D}$. (*c*) Momentum bandgap-edge phase variation and harmonics ($\omega_{o}$ and $\omega_{-1}$) mixing coefficient variation at different values of $M_{D}$. (*d*) The imaginary part of complex frequency variation within the momentum bandgap at $F_{s}/F_{m}=0.5$ ($F_{s}$ is the main signal frequency and $F_{m}$ is the modulation frequency) for harmonics $\omega_{o}$ and $\omega_{-1}$. At $M_{D}=0.66$, (*e*) unit cell electrical length variation with the real and imaginary parts (real is fixed at $F_{s}/F_{m}=0.5$ for imaginary part variation) of the complex frequency, (*f*) the imaginary part of complex frequency variation within the momentum bandgap at different real part values for harmonics $\omega_{o}$ and $\omega_{-1}$, (*g*) for harmonics $\omega_{o}$ and $\omega_{-1}$. The imaginary part of complex frequency variation with $M_{D}$ at $F_{s}/F_{m}=0.5$ and unit cell electrical length at nominal capacitance $C_{o}$ (βd=0.81). In the two Y-axis figures, dashed circles with arrows indicate the Y-axis utilized for the plotted curve.


A lossless lumped model TL loaded with a TMC is considered here. The dispersion diagram is plotted in figure 2*a* for the unit cell with series and shunt elements shown in figure 1*b*. To plot figure 2*a*, numerical values are chosen as follows: $F_{m}=1$ GHz (modulation frequency), L=12.5 nH, $C_{o}=5$ pF and $M_{D}=0.66$. The unit cell has a 50 Ω characteristic impedance at nominal capacitance $C_{o}$. Figure 2*a* shows that only the fundamental frequency $\omega_{s}$ exists in the absence of modulation; the red curves describe the forward and backward propagation dispersion at $\omega_{s}$ in case of no modulation. With modulation present, black curves describe the dispersion of six considered harmonics (*n* = 3) and the fundamental frequency. All curves are labelled with the harmonic order and type of propagation: forward (f) or backward (r). At $F_{s}=0.5$ GHz (main signal frequency), strong interaction happens between the fundamental $\omega_{s}$ and the harmonic $\omega_{-1}$ ($F_{m}=2F_{s}$), which operates at the same frequency. Due to the strong coupling between harmonics mentioned above, a region of unstable K-gap is created called momentum bandgap [1,29,36]. At this region, exponential amplification occurs for the signal, and the circuit acts as a parametric amplifier [37,38]. However, it is worth mentioning that the dispersion diagram is not affected by varying the modulation phase $\varphi_{M}$. As a result, contrary to parametric amplification, which is very sensitive to the phase matching between the pump and the main signals, no synchronization is needed between propagating and modulating signals in artificial time crystals [39–41] to achieve amplification. In lossless media (or circuits), two main factors affect the amplification level of time-modulated defined media (circuit): the modulation depth and the loading impedance. However, introducing loss to the system limits the amplification levels, as illustrated in §2.2.2 Figure 2*b* shows a close-up of the momentum bandgap region for different modulation depths. With a higher value of $M_{D}$, a wider momentum bandgap can be obtained. The phases at the edges of each bandgap are written to be compared with the unit cell’s electrical length, which is discussed in the following paragraph. Figure 2*c* shows the phase variation at the edges of the momentum bandgap with $M_{D}$. In the case of no-modulation ($M_{D}=0$), the momentum bandgap edges match in phase, and the momentum bandgap is closed. However, as $M_{D}$ increases, the momentum bandgap edges’ phases deviate away, indicating an increase in the gap width. Moreover, the phase deviation is not symmetric at both edges because only part of the TL is a time-variant component, which makes the relation between varying the TMC and unit cell Bloch impedance ($Z_{Bloch}$) nonlinear. Based on edges’ phase variation and analogy to mode coupling coefficient definition [42], the harmonics mixing coefficient (κ) is plotted in figure 2*c* following

(2.2) $$\kappa =\frac{{\left({Phase}_{edge1}\right)}^{2}-{\left({Phase}_{edge2}\right)}^{2}}{{\left({Phase}_{edge1}\right)}^{2}+{\left({Phase}_{edge2}\right)}^{2}}.$$

This quantity (κ) can be calculated from a dispersion diagram and serves as an indicator of the level of interaction between harmonics ($\omega_{0,-1}$) responsible for signal amplification. A higher value for this quantity indicates greater levels of integration and amplification. Figure 2*c* shows the relation between $M_{D}$ and κ. As expected, higher values of $M_{D}$ correspond to higher values of κ. Within the momentum bandgap, only complex frequencies associated with fundamental $\omega_{s}$ and the harmonic $\omega_{-1}$ exist and are plotted in figure 2*d*–*g*.

As shown figure 2*d*, an imaginary dispersion diagram is plotted considering harmonics ($\omega_{0,-1}$) at different values of $M_{D}$ with a fixed real frequency $F_{s}=0.5$ GHz (main signal frequency) and varying the imaginary part. The positive imaginary frequency causes the signal growth, while the negative imaginary frequency is responsible for the signal decay. As shown, there is a direct relation between imaginary part values, the momentum bandgap width and the value of $M_{D}$. Within the momentum bandgap, as $M_{D}$ increases, the strength of the harmonic ($\omega_{-1}$) and the imaginary frequency increase. Consequently, at a sufficient value of $M_{D}$, signal growth starts. This sufficient value of $M_{D}$ depends on the cells’ formation, their number, the matching at the terminals (load and source) and (modulation frequency) ($F_{m}$). Both real and imaginary ($F_{s}=0.5$ GHz as real part) dispersion diagrams are plotted in figure 2*e* at $M_{D}=0.66$ to show that imaginary frequencies only exist within the momentum bandgap. Deviating from the frequency real part value of $F_{s}=0.5$ GHz, the imaginary part gradually vanishes due to the existence of real frequency solutions of the eigenvalue problem, as shown in figure 2*f*. In other words, pure-time modulating the media causes the creation of momentum bandgaps at a sole frequency ratio of $F_{m}=2F_{s}$ [43] where only an imaginary frequency solution exists. Now, focusing on the unit cell electrical length at $C_{o}$ (0.81 rad), imaginary part variation of the fundamental $\omega_{s}$ and the harmonic $\omega_{-1}$, at fixed real part of $F_{s}=0.5$ GHz value, is plotted in figure 2*g* with $M_{D}$. As expected, the harmonic $\omega_{-1}$ imaginary part is directly proportional to the value of $M_{D}$.

**Figure 3.**
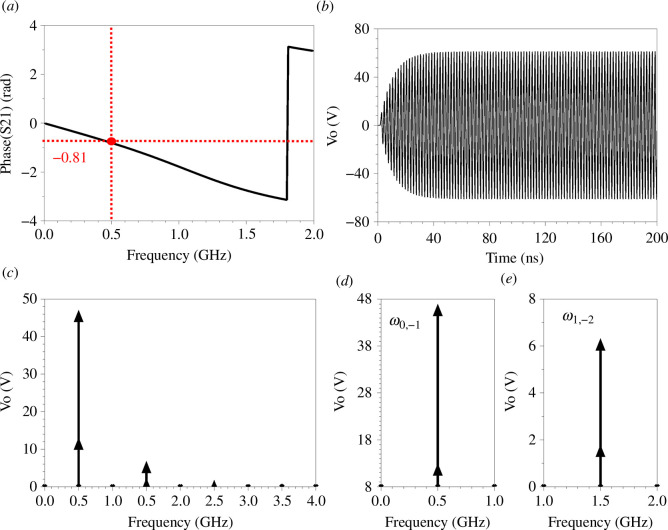
(*a*) Phase of $S_{21}$ for only one unit cell shown in figure 1*b* with L=12.5 nH and $C_{o}=5$ pF (static), S-parameter simulation. For nine unit cells with 10 V input peak (1 W (RMS)), TMC at $M_{D}=0.66$, L=12.5 nH, $C_{o}=5$ pF, $F_{s}=0.5$ GHz (main signal frequency), $F_{m}=1$ GHz (modulation frequency) loaded with a 50 Ω load impedance. (*b*) Output voltage (transient). (*c*) Output voltage (harmonic balance). (*d*) Close-up look for the harmonics at 0.5 GHz. (*e*) Close-up look for the harmonics at 1.5 GHz.

S-parameter, transient and harmonic balance simulations in ADS are utilized for further investigation. Figure 3*a* shows the phase of $S_{21}$ of the unit cell shown in figure 1*b* with L=12.5 nH and C=5 pF (static), done by S-parameter simulation. The unit cell has an absolute electrical length of 0.81 radians at $F_{s}=0.5$ GHz (main signal frequency). As expected, this phase lies within the momentum bandgap illustrated in figure 2.

Transient and harmonic balance simulations were conducted on nine unit cells with TMC at ($M_{D}=0.66$) and ($F_{m}=1$) GHz, truncated with a 50 Ω load impedance. Figure 3*b* illustrates the transient simulation with a 10 V (peak) matched source (1 W (RMS)) at ($F_{s}=0.5$) GHz. Notably, 50 Ω matched voltage sources were used, connected in series with an ideal 50 Ω isolator. The output power after the isolator is 1 W (RMS), and the output voltage is 10 V peak. Within a 200 ns simulation period, the output voltage is amplified, reaching a 65 V peak level. For the same circuit, harmonic balance results are shown in figure 3*c*–*e*. Figure 3*d*,*e* provide close-up views of the harmonics of interest. Two amplified harmonics are observed at 0.5 GHz (*n* = 0,–1) and another two harmonics (n = −2,1) at 1.5 GHz. There is agreement between the simulated amplification results obtained using harmonic balance and transient simulations. The output amplified voltage from the transient simulation is almost equal to the summation of the two harmonics (*n* = 0,–1) at 0.5 GHz obtained from the harmonic balance simulation. Typically, transient simulations are more accurate than harmonic balance simulations in highly nonlinear circuits. However, the harmonic balance results show the output harmonics, which is useful for verifying the results depicted in figure 2.

**Figure 4.**
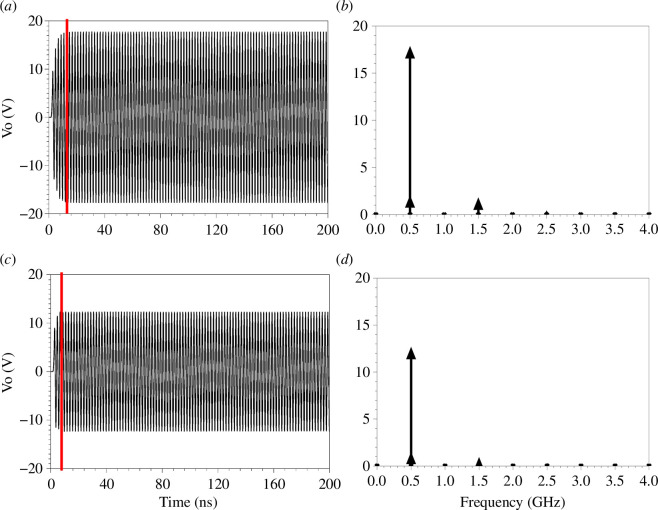
For nine unit cells (figure 1*b*) with 10 V input peak (1 W (RMS)), L=12.5 nH, $C_{o}=5$ pF, $F_{s}=0.5$ GHz (main signal frequency), $F_{m}=1$ GHz (modulation frequency), loaded with a 50 Ω load impedance. (*a*) Output voltage (transient) at TMC at $M_{D}=0.5$. (*b*) Output voltage (harmonic balance) at TMC with $M_{D}=0.5$. (*c*) Output voltage (TS) at TMC with $M_{D}=0.33$. (*d*) Output voltage (harmonic balance) at TMC with $M_{D}=0.33$.

For lower values of ($M_{D}$), figure 4*a*,*b* and *c*,*d* show the simulation results at ($M_{D}=0.5$) and ($M_{D}=0.33$), respectively. At ($M_{D}=0.5$), the output voltage (transient, figure 4*a*) saturates after 12 ns at 18 V (peak), which is close to the summation of the two harmonics (harmonic balance, figure 4*b*) at 0.5 GHz. At ($M_{D}=0.33$), the output voltage (transient, figure 4*c*) saturates after 8 ns at 13 V, which is exactly the summation of the two harmonics (harmonic balance, figure 4*d*) at 0.5 GHz.

**Figure 5.**
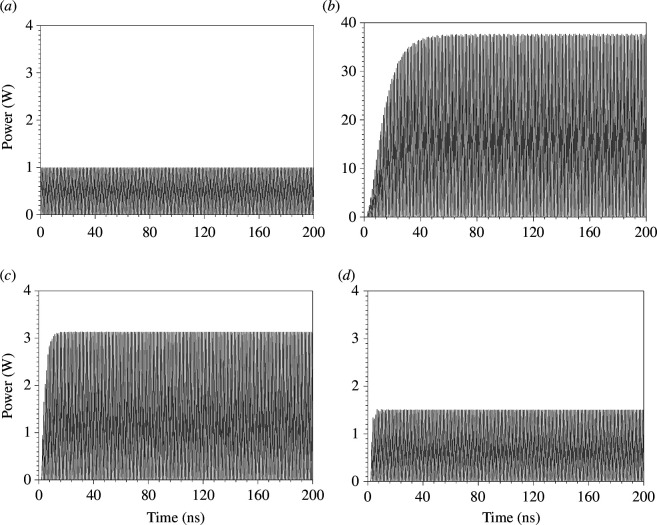
For nine unit cells (figure 1*b*) with 10 V input peak (1 W (RMS)), L=12.5 nH, $C_{o}=5$ pF, $F_{s}=0.5$ GHz (main signal frequency), $F_{m}=1$ GHz (modulation frequency), loaded with a 50 Ω load impedance. (*a*) Input instantaneous power (RMS) in the absence of modulation (transient). Transient simulation output power (RMS) with the TMC at (*b*) $M_{D}=0.66$, (*c*) $M_{D}=0.5$ and (*d*) $M_{D}=0.33$.

Figure 5 illustrates the transient instantaneous output powers under various conditions. In figure 5*a*, the input power is set at 1 W, a value consistently used throughout the manuscript. When employing the time-modulated capacitor, the instantaneous output power is amplified. An increase in the modulation depth value results in higher output power at the load. As depicted in figure 5*b*–*d*, the output average power saturates at 37, 3 and 1.4 W for modulation depths of ($M_{D}=0.66$), ($M_{D}=0.5$) and ($M_{D}=0.33$), respectively. These simulation results confirm that power amplification is directly proportional to the modulation depth ($M_{D}$).

### 2.2. Signal amplification in a lossless transmission line loaded with time-modulated capacitor

To investigate a lossless TL loaded with TMC and the effect of varying the cell electrical length, the element (A) in figure 1*a* is replaced by a TL section with an electrical length of 2mπ radian at the modulation frequency and 90 Ω characteristic impedance ($Z_{o}$). Two conditions are considered for element (B) in figure 1*a*: lossless TMC and lossy TMC.

#### 2.2.1. Lossless time-modulated capacitor

In this subsection, element (B) will be a lossless TMC. The considered series and shunt elements can be seen in figure 1*c*, and different cases are investigated at various values of m.

**Figure 6.**
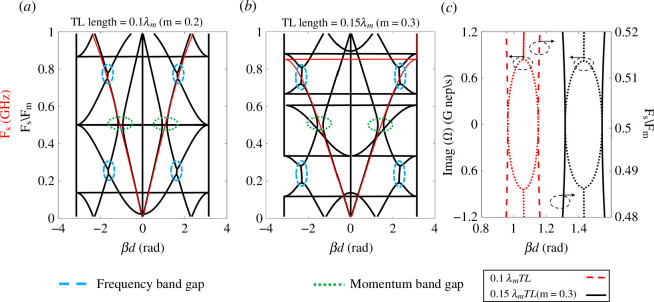
Dispersion diagram of the unit cell in figure 1*c* with $C_{o}=4$ pF, $M_{D}=0.5$, 90 Ω characteristic impedance ($Z_{o}$), and TL sections with different lengths. (*a*) TL length $0.1\lambda_{m}$ (wavelength at modulation frequency), and (*b*) TL length $0.15\lambda_{m}$ (m=0.15). (*c*) Close-up look of the momentum bandgaps and complex frequencies.

Adding TL to the unit cell in figure 1*a* as element (A) allows us to investigate the effect of varying the electrical length of the TL on the amplification. Changing the electrical length of the time-modulated unit cell will allow the control of the location of the frequency bandgaps in the dispersion diagram of one of the prominent harmonics ($\omega_{0,-1}$) relative to the momentum bandgap (time). The dispersion diagram (*n* = 3) of the unit cell shown in figure 1*c* is plotted in figure 6 for different lengths of the TL. 90Ω $Z_{o}$ TL sections are utilized to match the unit cell $Z_{Bloch}$ at nominal capacitance $C_{o}$ to a 50 Ω load. Red curves in figure 6 represent the dispersion curve of the unit cell without any modulation. In the case of no-modulation, it can be seen that varying the TL length changes the position of the frequency bandgap that normally starts from and ends by phase ±π [34]. With time modulation, the momentum bandgap appears at $F_{s}=0.5$ GHz, considering $F_{m}=1$ GHz; green circles note the momentum bandgaps. In addition, due to the interaction between harmonics, frequency bandgaps are created at phase values different from ±π. The frequency bandgaps are noted in blue circles. We are interested in frequency bandgaps occurring by one of the prominent harmonics ($\omega_{0,-1}$). As shown in figure 6, as the frequency bandgap becomes closer to the momentum bandgap, the momentum bandgap becomes wider and the slope of the dispersion curves around the momentum bandgap increases. Despite the expansion of the momentum bandgap, the values of the imaginary part of the frequency remain nearly identical, as illustrated in figure 6*c*. Thus, increasing the cell electrical length by extending the TL length, up to a certain limit without undermining the integrity of the pure time-modulation profile, broadens the momentum band. Interestingly, this broadening is not followed by a corresponding increase in the values of the imaginary part of the frequency. Instead, it signifies an increase in the phase range where complex frequencies exist, leading to enhanced amplification.

**Figure 7.**
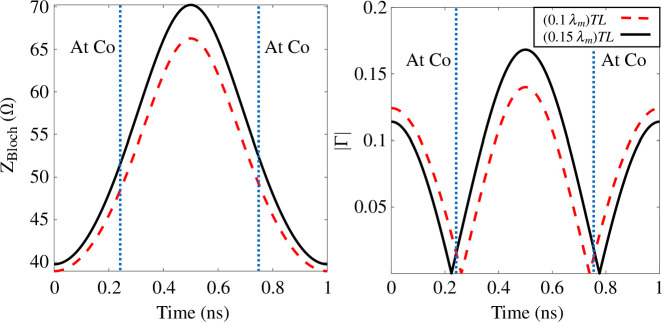
For unit cell in figure 1*c* with $C_{o}=4$ pF, $M_{D}=0.58$, Ω characteristic impedance ($Z_{o}$), TL section lengths $0.1\lambda_{m}$ (wavelength at modulation frequency) and $0.15\lambda_{m}$, at the fundamental frequency (0.5 GHz). (*a*) Variation in Bloch impedances across time for one complete cycle. (*b*) The absolute value of the reflection coefficient (|Γ|) within one complete cycle considering 50 Ω load (source).


To further investigate the effect of TL length on the momentum bandgap width, the variation of the unit cell Bloch impedances with time are plotted in figure 7 for the fundamental frequency of 0.5 GHz. As in [34], Bloch impedance can be plotted using

(2.3) $$Z_{Bloch}^{\pm }=\frac{-2B}{A-D\mp \sqrt{{\left(A+D\right)}^{2}-4}},$$

where A, B and D are elements in the ABCD matrix of the unit cell at the fundamental frequency ($\omega_{s}$). The ± solutions correspond to $Z_{Bloch}$ for forward and backward travelling waves. Figure 7*a* shows the Bloch impedance variation for the unit cell of TL lengths $0.1\lambda_{m}$ (wavelength at modulation frequency) and $0.2\lambda_{m}$ sections within one complete cycle. It can be seen that a unit cell of $0.2\lambda_{m}$ TL section has a slightly wider impedance variation range of 41–70 Ω compared with the unit cell of $0.1\lambda_{m}$ TL section, which shows 40–66 Ω impedance variation range. This agrees with the results shown in figure 6; a unit cell of $0.15\lambda_{m}$ TL section has a wider momentum bandgap than the unit cell of $0.1\lambda_{m}$ section. As $M_{D}$ increases, a wider range of impedances and momentum bandgaps are also expected. In addition, the difference between unit cells of $0.1\lambda_{m}$ and $0.15\lambda_{m}$ TL sections in the momentum bandgap and impedance variation range becomes more apparent. Figure 7*b* shows the absolute value of the reflection coefficient (|Γ|) within one complete cycle considering a 50 Ω load (source), which is computed using


(2.4) $$\Gamma_{load\;(source)}=\frac{Z_{load\;(source)}-Z_{Bloch}}{Z_{load\;(source)}+Z_{Bloch}}.$$

As shown in figure 7*b*, the values of |Γ| are almost the same except at the half of the cycle, where |Γ| is higher for $0.2\lambda_{m}$ TL unit cell compared with $0.1\lambda_{m}$ TL unit cell. In a pure-time modulated medium (where the unit cell exhibits minimal phase variation with respect to the input signal, and all modulated capacitors are synchronized), the dispersion diagram is identical for both forward and backward propagation, as shown in figure 6. However, if there are high mismatches at the terminals, all reflected signals will be re-amplified during backward propagation, leading to further reflections at the source terminal. Eventually, the transmission line becomes unstable and causes signal oscillation [43]. Also, Increasing |Γ| slightly could enhance the amplification for $0.2\lambda_{m}$ TL unit cell because the reflected signal can be added constructively to the main signal and re-amplified. However, the circuit will be more sensitive to the phase difference between propagating signals and modulation profile, as discussed in §3.

**Figure 8.**
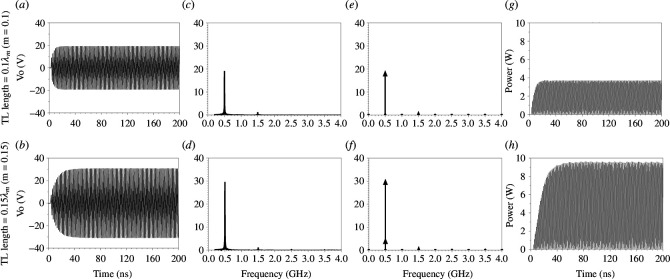
For nine unit cells (figure 1*c*) with 10 V input peak (1 W (RMS)), $C_{o}=4$ pF $F_{s}=0.5$ GHz, $F_{m}=1$ GHz, $M_{D}=0.58$, and the TL with 90 Ω characteristic impedance ($Z_{o}$) and different lengths loaded with a 50 Ω load impedance. For $0.1\lambda_{m}$ (m=0.1) TL: (*a*) output voltage (transient), (*c*) Fourier transform (FT) of the output voltage (transient), (*e*) output voltage (harmonic balance), and (*g*) power output (transient). For $0.15\lambda_{m}$ (m=0.15) TL: (*b*) output voltage (transient), (*d*) FT of the output voltage (transient), (*f*) output voltage (harmonic balance) and (*h*) power output (transient).

Transient simulations were conducted for nine unit cells (figure 1*c*) with a 50 Ω matched source supplying 10 V peak (1 W), $C_{o}=4$ pF, $F_{s}$ = 0.5 GHz, $F_{m}$ = 1 GHz, $M_{D}=0.58$ and a TL with a 90 Ω characteristic impedance ($Z_{o}$) of varying lengths, loaded with a 50 Ω load impedance. Two TL lengths were chosen ($0.1\lambda_{m}$ and $0.15\lambda_{m}$) to be nearly matched at $C_{o}$. The results, shown in figure 8, indicate signal amplification.

The unit cell consists of ideal elements, and introducing loss would cause the circuit to saturate at lower values, affecting the system’s responsiveness to modulation. Figure 8*a*,*b* display the transient simulations for TL lengths of $0.1\lambda_{m}$ and $0.15\lambda_{m}$, respectively. Figure 8*c*,*d* show the Fourier transforms of the results obtained in figure 8*a*,*b*. As the TL length increases, higher amplification values are achieved; the output voltage is amplified to 18 and 28 V using TL lengths of $0.1\lambda_{m}$ and $0.15\lambda_{m}$, respectively.

Figure 8*e*,*f* present the harmonic balance simulation results, which align with the transient results shown in figure 8*a*,*b*. The input power remains the same as shown in figure 5*a*. The instantaneous output power at TL lengths of $0.1\lambda_{m}$ and $0.15\lambda_{m}$ with modulation are shown in figure 8*g*,*h*, respectively. The instantaneous output power is amplified to 3.5 and 9.2 W using TL lengths of $0.1\lambda_{m}$ and $0.15\lambda_{m}$, respectively.

The simulation results correspond with the analysis using the dispersion diagram and Bloch impedance. Compared with the unit cell with a $0.1\lambda_{m}$ TL section, the unit cell with a $0.15\lambda_{m}$ TL section has a wider momentum bandgap and a greater variation range of Bloch impedances. Consequently, the unit cell with a $0.15\lambda_{m}$ TL section exhibits higher voltage and power amplification values.

#### 2.2.2. Lossy time-modulated capacitor

In this subsection, element (A) in figure 1*a* is a TL with length $0.15\lambda_{m}$ (m = 0.15) and 90 Ω characteristic impedance ($Z_{o}$). Element (B) will be a TMC with series resistance Rc, as illustrated in figure 1*d*. The introduced resistance Rc adds loss to the TMC, and the unit cell will be studied at different Rc values.

**Figure 9.**
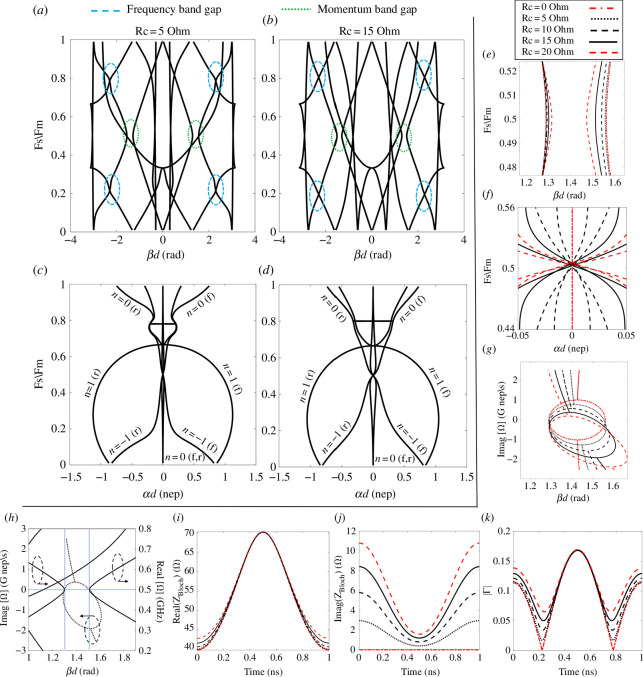
For the unit cell in figure 1*d* with $C_{o}=4$ pF, $M_{D}=0.58$, and a TL with 90 Ω characteristic impedance ($Z_{o}$) and length of $0.15\lambda_{m}$ (m = 0.15). dispersion diagram at (*a*) Rc = 5 Ω and (*b*) Rc = 15 Ω. Attenuation variation versus $F_{s}/F_{m}$ at (*c*) Rc = 5 Ω and (*d*) Rc = 15 Ω. For various values of Rc (0, 5, 10, 15, 20) Ω, (*e*) Close-up look of the momentum bandgap, (*f*) The imaginary part of complex frequency variation within the momentum bandgap at $F_{s}/F_{m}=0.5$ for harmonics $\omega_{o}$ and $\omega_{-1}$ and (*g*) Close-up look of attenuation at the momentum bandgap. (*h*) For Rc = 15 Ω, the unit cell electrical length variation with the real and imaginary parts (real is fixed at $F_{s}/F_{m}=0.5$ for imaginary part variation) of the complex frequency. (*i*) The real part of Bloch impedance variation within one complete cycle. (*j*) The imaginary part of the Bloch impedance variation within one complete cycle. (*k*) The absolute value of the reflection coefficient (|Γ|) within one complete cycle considering 50 Ω load (source). In the two Y-axis figures, dashed circles with arrows indicate the Y-axis utilized for the plotted curve.

The dispersion diagrams are plotted in figure 9 utilizing (S16) and (S23) in the electronic supplementary material. In figure 9*a*,*b*, the dispersion diagram is plotted at Rc values of 5 and 15 Ω, respectively. Compared with figure 9*b*, weak interaction between harmonics can be observed in the areas of the frequency bandgaps, circled in blue, and the momentum bandgaps, circled in green. As the value of Rc increases, the frequency bandgap and momentum bandgap areas shrink. Moreover, to further investigate the effect of Rc, the attenuation constant variations with frequency associated with the cases shown in figure 9*a*,*b* are shown in figure 9*c,d*, respectively. Focusing on the prominent harmonics ($\omega_{0,-1}$), the attenuation constant increases at all frequencies as the Rc value increases except for $F_{s}=2F_{m}$; attenuation is always zero as long as the momentum bandgap exists. To clearly show the effect of increasing the value of Rc on the momentum bandgap and attenuation, a close-up look of the momentum bandgap and attenuation at $F_{s}=2F_{m}$ at different values of Rc are plotted in figure 9*e*,*f*, respectively. As shown in figure 9*e*, the momentum bandgap shrinking is obvious as an effect of increasing the value of Rc. On the other hand, as shown in figure 9*f*, despite hitting the zero value of attenuation at $F_{s}=2F_{m}$ regardless of the value of Rc, attenuation at other frequencies increases with the increase of Rc. It is worth mentioning that if Rc reaches the value that closes the momentum bandgap, attenuation will not be zero any more at $F_{s}=2F_{m}$. In figure 9*g*, an imaginary dispersion diagram is plotted at different values of Rc considering harmonics ($\omega_{0,-1}$) with a fixed real frequency $F_{s}=0.5$ GHz. As Rc increases, the imaginary frequency moves more to the negative region, explaining the expected drop in amplification levels. In figure 9*h*, real and imaginary (real at $F_{s}=0.5$) dispersion diagrams are plotted at Rc = 15 Ω. The momentum bandgap width is mainly defined by the phases at which the positive imaginary frequency exists. In this case (Rc = 15 Ω) and within the momentum bandgap, the imaginary frequency takes the values confined between the vertical blue dotted lines because, outside this region, real solutions to the eigenvalue problem exist.

Figure 9*i*–*k* illustrate the effect of $R_{c}$ on the Bloch impedance ($Z_{Bloch}$) and |Γ| at the terminals. As $R_{c}$ increases, the variation range of the real part of $Z_{Bloch}$ diminishes (figure 9*i*). Additionally, the imaginary part of $Z_{Bloch}$ rises, indicating an increase in loss within the unit cell (figure 9*j*). Furthermore, the circuit gradually loses matching at the 50 Ω terminals at the quarter cycle (nominal $C_{o}$) as $R_{c}$ increases (figure 9*k*). Therefore, it can be concluded that the value of $R_{c}$ is inversely proportional to the signal amplification gain.

**Figure 10.**
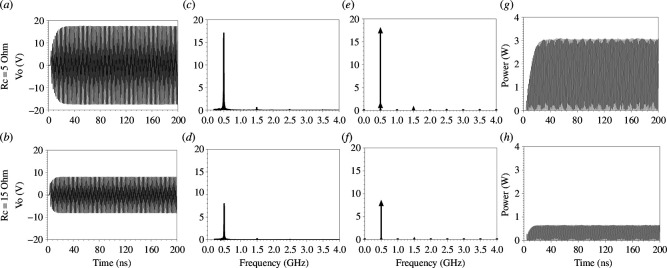
For nine unit cells (figure 1*d*), with 10 V input peak (1 W (RMS)), $C_{o}=4$ pF, $F_{s}=0.5$ GHz, $F_{m}=1$ GHz, $M_{D}=0.58$, a TL with 90 Ω characteristic impedance ($Z_{o}$) and length of $0.15\lambda_{m}$ loaded with a 50 Ω load impedance. (*a, b*) Voltage output (transient) at Rc = 5 and 15 Ω, respectively. (*c, d*) Fourier transforms for transient results at Rc = 5 and 15 Ω, respectively. (*e, f*) Voltage output (harmonic balance) at Rc = 5 and 15 Ω, respectively. (*g, h*) Power output (transient) at Rc = 5 and 15 Ω, respectively.

To confirm the observed weak interaction between harmonics and its relation to Rc, transient simulation is performed with nine unit cells (figure 1*d*) with a 50 Ω matched source supplying 10 V peak (1 W), $C_{o}=4$ pF, $F_{s}=0.5$ GHz, $F_{m}=1$ GHz, $M_{D}=0.58$, a TL with 50 Ω characteristic impedance ($Z_{o}$) and length of $0.15\lambda_{m}$ loaded with a 50 Ω load impedance. Two values of Rc are considered, 5 and 15 Ω, and the results are plotted in figure 10. For Rc = 5 Ω, the output voltage saturates at the peak of 17 V (figure 10*a*,*c*,*e*), and the instantaneous output power saturates at 3 W (figure 10*g*). Increasing the value of Rc to 15 Ω causes a drop in the output voltage to 7 V peak value (figure 10*b*,*d*,*f*), and in the instantaneous output power to 0.6 W (figure 10*h*). As shown, when Rc reaches 15 Ω, there is no amplification.

## 3. Signal-modulation phase sensitivity and practical consideration

The dispersion diagrams depicted in figures 2, 6 and 9 remain unaffected by the modulation phase ($\varphi_{M}$), defined in equation (2.1). This characteristic presents a significant advantage of signal amplification in time-modulated lines over that in parametric amplifiers. In a sensitive-parametric amplifier operating under the condition $f_{p}=2f_{s}$, the amplification process involves mixing the primary and the pump signals using a nonlinear device. As a result, the gain achieved is highly susceptible to the phase difference between the pump and primary signals [44]. Contrastingly, the ideal approach in time-modulated media realization involves the use of linear devices. This method ensures that the amplification results primarily from the time-modulation effect, rather than a mixture of time-modulation and parametric amplification. Signal-modulation phase difference ($\Delta \varphi_{S-M}$) serves as a key indicator of such a combination. A smaller value of ($\Delta \varphi_{S-M}$) suggests that the amplification is predominantly due to the time-modulation effect, and vice versa.

**Figure 11.**
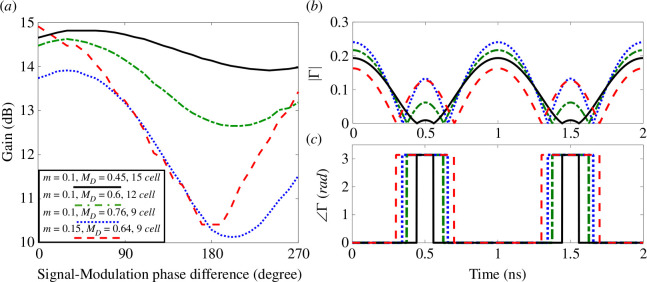
At different transmission line lengths (figure 1*c*), $M_{D}$ values and the number of cells (10 V input peak (1 W (RMS)), $F_{s}=0.5$ GHz, $F_{m}=1$ GHz, 50 Ω load): (*a*) output power gain versus phase difference between the input signal and modulated capacitors ($\Delta \varphi_{S-M}$), (*b*) the absolute value of the reflection coefficient, (*c*) the angle of the reflection coefficient. (Each cell has $C_{o}=4$ pF and a TL with 90 Ω characteristic impedance ($Z_{o}$).)

One factor that contributes to the degradation of signal-modulation phase insensitivity in time-modulated media is the truncation with static media, which leads to mismatches and reflections at the terminals. To explore the impact of dynamic mismatches at the terminals, we examine different transmission line lengths (figure 1*c*), $M_{D}$ values and cell counts. The output power gain is plotted versus the phase difference between signal and modulation ($\Delta \varphi_{S-M}$) in figure 11*a*. Starting with a close value of the gain (approx. 14 dB) at $\Delta \varphi_{S-M}=0$, various combinations of transmission line lengths (m), $M_{D}$ values, and cells counts are chosen. As demonstrated in §2.2, for the same number of cells, cells with m=0.1 TL (represented by blue curves) require a higher value of $M_{D}$ (=0.76) to achieve a power gain comparable to cells with m=0.15 TL (represented by red curves). However, when $\Delta \varphi_{S-M}$ varies, cells with m=0.15 TL exhibit greater power gain instability compared with cells with m=0.1 TL. To understand this, the absolute value and the phase of reflection coefficient variation over time, considering 50 Ω load, are plotted in figure 11*b*,*c*, respectively. Despite similar absolute values of Γ within the modulation cycle period, cells with m=0.15 TL show a broader period of flipping Γ angle sign (to ∠Γ=π) compared with cells with m=0.1 TL. To reduce the values of |Γ| and the periods of flipping ∠Γ, gain variations for cells with m=0.1 TL are plotted for lower $M_{D}$ values and higher number of cells. Employing lower $M_{D}$ values helps minimize the periods of flipping ∠Γ (to ∠Γ=π) and the values of |Γ| within these periods. Consequently, the signal-modulation phase insensitivity in time-modulated transmission lines is improved. Flipping the sign of the reflection coefficient enhances the constructive and destructive interference between forward and backward propagating waves, which is highly sensitive to $\Delta \varphi_{S-M}$. Therefore, to maintain gain stability, it is more effective to increase the number of cells rather than the $M_{D}$ values.

**Figure 12.**
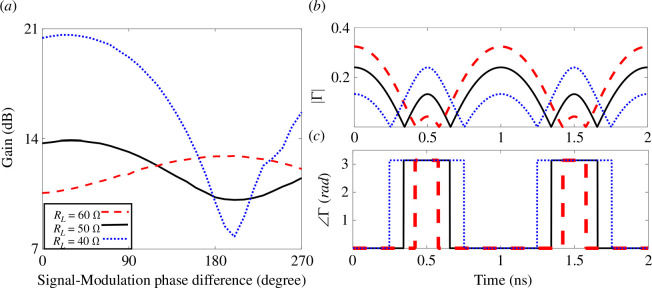
For nine unit cells (figure 1*c*), with 10 V input peak (1 W (RMS)), and length of $0.1\lambda_{m}$ (m=0.1) and $M_{D}=0.76$ at different load impedances: (*a*) output power gain versus phase difference between the input signal and modulated capacitors ($\Delta \varphi_{S-M}$), (*b*) the absolute value of the reflection coefficient, (*c*) the angle of the reflection coefficient. (Each cell has $C_{o}=4$ pF and a TL with 90 Ω characteristic impedance ($Z_{o}$).)

The signal-modulation phase insensitivity is also tested for various load impedance values, as shown in figure 12. For a configuration of nine cells with m=0.1 TL, $M_{D}=0.76$, and a nominal Bloch impedance of 50 Ω, the output power gain variations with $\Delta \varphi_{S-M}$ are plotted for 60,50 and 40 Ω loads in figure 12*a*. Additionally, the absolute value and the phase of the reflection coefficient variation over time for these loads are depicted in figure 12*b*,*c*, respectively. The same observations can be obtained. Utilizing a load that helps minimize the periods of flipping ∠Γ and the values of |Γ| within these periods will enhance signal-modulation phase insensitivity. For a desired value of load impedance and high signal-modulation phase insensitivity, the cells can be designed to achieve minimum periods of flipping ∠Γ and the values of |Γ| within these periods.

To implement time-modulated TL, three primary considerations must be taken into account. Firstly, the operation should be within the linear regime of the switchable device under consideration. Secondly, the switchable device should possess a high-speed response to keep pace with the high-frequency control signal. Lastly, effective isolation should be ensured between the propagating and control signals. The photodiode is a potential candidate device [45,46]. It offers switchability between two states, high-speed responsivity, and effective isolation between the control (light) and propagating signals. However, the set-up is complex, costly and susceptible to various types of noise such as thermal, shot and avalanche noise. The Schottky diode is another viable option [47]. Despite its high-speed responsivity, the diode is a two-port network. It is crucial to operate within its linear region to minimize the mixing between control and propagating signals. Moreover, it is preferable to use it for amplifying weak signals below the diode’s threshold voltage, to ensure the diode is controlled only by the control signal. One potential application for using the Schottky diode in time-modulated transmission lines is to amplify the qubit read-out signal in quantum computing [48]. The read-out signal is weak, and time-modulated transmission lines are immune to phase noise from the control signal local oscillator compared with the parametric amplifier [44]. Furthermore, the Schottky diode is a simple junction that can be fabricated in a manner that minimizes semiconductor noises [47].

## 4. Conclusion

Signal growth in a TL with time-modulated $Z_{o}$ is investigated by studying the eigenvalue problem and confirmed by circuit modeling. Three models are considered: a lossless L-C TL lumped model with a shunt time-modulated capacitor, a TL loaded with a shunt lossless time-modulated capacitor, and a TL loaded with shunt lossy time-modulated capacitor to study the loss effect. The eigenvalue problem is discussed in detail, and the real and imaginary dispersion diagrams are plotted for each model. Modulation, the Bloch impedance of the unit cell and the reflection coefficient at the terminals are plotted and discussed. S-parameters, transient and harmonic balance simulations are performed, and the results are consistent with the dispersion diagram, Bloch impedance variation of the unit cell, and reflection coefficient variation at the terminals. The third model discusses the loss effect by plotting the attenuation variation with frequency and real and imaginary dispersion diagrams. In a lossy time-modulated media, if the loss is low enough to allow the momentum bandgap to be created at $F_{s}=2F_{m}$, attenuation will be zero at $F_{s}=2F_{m}$. However, the drop in amplification level happens because of the movement of imaginary frequency within the momentum bandgap to the negative region, causing the signal decay. Ideally, the amplification in a time-modulated line should not be affected by variation of signal-modulation phase difference. However, truncating time-modulated transmission with a static load can degrade signal-modulation phase insensitivity in time-modulated media. This phenomenon is studied and discussed.

## Data Availability

Data is available online [49]. Two main folders exist: Datasets It contains the Excel files generated from the transient time simulator and plotted in the manuscript (named according to the manuscript). Code/Software It contains two folders. The first contains the MATLAB codes for all proposed models. They are used to plot the real and complex frequency dispersion and the loss dispersion. The second is the simulator file (Advanced Design System) that is used to confirm all the results obtained from dispersion plots. Matlab files: All folders have (eigenshuffle.m) that helps to arrange the eigenvalues coming out from the solution of the eigenvalue problem. -L_C_(section3): contains (L_C_Beta.m) that plots the real dispersion for the modulated TL lumped model, and (L_C_imagfre.m) that plots the complex dispersion for modulated TL lumped model. -TL_C_(section4): contains (TL_RC_Beta.m) that plots the real dispersion for modulated TL loaded with shunt capacitor (Lossy or lossless by setting the value of Rc), and (TL_RC_imagfre.m) that plots the complex dispersion for modulated TL loaded with shunt capacitor (Lossy or lossless by setting the value of Rc), and (TL_RC_ALFA.m) that plots the loss dispersion for modulated TL loaded with shunt capacitor (Lossy or lossless by setting the value of Rc). ADS File: It contains three schematics for the proposed three models with transient and harmonic balance simulations that confirm the results obtained from the solution of the eigenvalue problem. Supplementary material is available online [50].
